# A Single-Field Finite Difference Time-Domain Method Verified Using a Novel Antenna Design with an Artificial Magnetic Conductor Enhanced Structure

**DOI:** 10.3390/mi16040489

**Published:** 2025-04-21

**Authors:** Yongjun Qi, Weibo Liang, Yilan Hu, Liang Zhang, Cheng You, Yuxiang Zhang, Tianrun Yan, Hongxing Zheng

**Affiliations:** 1 School of Computer Science and Engineering, North China Institute of Aerospace Engineering, Langfang 065000, China; qyj@nciae.edu.cn (Y.Q.); zhangliang@nciae.edu.cn (L.Z.); 2 Hebei Key Laboratory of Optical Fiber Biosensing and Communication Devices, School of Information Engineering, Handan University, Handan 056005, China; wbliang@foxmail.com; 3 School of Electronics and Information Engineering, Hebei University of Technology, Tianjin 300401, China; yilan_hu@yeah.net (Y.H.); youcheng_hbt@163.com (C.Y.); zhangyuxiang_hbt@163.com (Y.Z.); yantianrun_hbt@foxmail.com (T.Y.)

**Keywords:** single-field Finite Difference Time-Domain, hybrid implicit–explicit, weakly conditional stability, dual-band antenna, artificial magnetic conductor, artificial intelligence optimization

## Abstract

The Finite Difference Time-Domain (FDTD) method is a powerful tool for electromagnetic field analysis. In this work, we develop a variation of the algorithm to accurately calculate antenna, microwave circuit, and target scattering problems. To improve efficiency, a single-field (SF) FDTD method is proposed as a numerical solution to the time-domain Helmholtz equations. New formulas incorporating resistors and voltage sources are derived for the SF-FDTD algorithm, including hybrid implicit–explicit and weakly conditionally stable SF-FDTD methods. The correctness of these formulas is verified through numerical simulations of a newly designed dual-band wearable antenna with an artificial magnetic conductor (AMC) structure. A novel antenna fed by a coplanar waveguide with a compact size of 15.6 × 20 mm^2^ has been obtained after being optimized through an artificial intelligent method. A double-layer, dual-frequency AMC structure is designed to improve the isolation between the antenna and the human body. The simulation and experiment results with different bending degrees show that the antenna with the AMC structure can cover two frequency bands, 2.4 GHz–2.48 GHz and 5.725 GHz–5.875 GHz. The gain at 2.45 GHz and 5.8 GHz reaches 5.3 dBi and 8.9 dBi, respectively. The specific absorption rate has been reduced to the international standard range. In particular, this proposed SF-FDTD method can be extended to analyze other electromagnetic problems with fine details in one or two directions.

## 1. Introduction

The Finite Difference Time-Domain (FDTD) method, proposed by K.S. Yee in 1966 [1], has been widely applied in research and engineering for acoustic, microwave, and optical simulations. The FDTD method solves Maxwell’s equations in a wide variety of electromagnetic structures, including inhomogeneous, nonlinear, and frequency-dependent media [2]. Its parallelism and ability to obtain frequency-domain responses via Fourier Transform make it one of the most attractive methods for electromagnetic analysis.

Extensive work has been done to improve the FDTD method. The time step is restricted by the spatial step, leading to inefficiency. To address this, unconditionally or weakly conditionally stable FDTD methods have been developed employing both explicit and implicit schemes to relax the Courant–Friedrichs–Lewy (CFL) condition. Explicit unconditionally stable FDTD algorithms include the Associated Hermite (AH) method [3] and others [4], while implicit methods include the Alternating Direction Implicit (ADI) [5], Newmark-beta [6], and single-field (SF) [7] methods.

Recently, to accommodate parallel and multi-threaded computing, the SF-FDTD algorithm, introduced in [8], has been used to solve two-dimensional (2-D) Maxwell’s equations by updating only the electric (E-) fields, forming a representation of the time-domain Helmholtz wave equation. Subsequent work extended this to hybrid implicit–explicit (HIE) [9], weakly conditionally stable (WCS) [10], and 3D unconditionally stable [11] SF-FDTD algorithms. In this method, the iteration of the magnetic (H-) field is optional, allowing for E-field-only updates, which reduces the number of equations, accelerates simulation speed, and saves memory. This is advantageous for simulating microstrip circuits and other electromagnetic problems where H-field updates are not essential.

The classical FDTD method refers to the original Yee-algorithm-based approach [1], which discretizes Maxwell’s equations using staggered grids and explicit time stepping under the strict Courant–Friedrichs–Lewy (CFL) stability condition. However, this method does not solve all problems, and, because the code is not open, many engineering problems also need to be solved with the help of engineering software, but software is very expensive, and we have to research and develop it according to our own needs as an important supplement to engineering software.

To apply the proposed SF-FDTD code above, there are some examples, such as microwave circuits, antennas, and target scattering, in real applications. In fact, broadband antennas are increasingly favored in modern wireless communication systems due to their ability to adapt to limited spectrum resources and antenna size constraints. Circularly polarized antennas reduce multipath fading effects, and multi-layer structures optimize bandwidth by reducing the Q-factor [12].

The improvement of their medical uses has expanded the application range of antennas working in the Industrial, Scientific, and Medical (ISM) dual band, making it easier to achieve device interconnection. However, people’s pursuit of quality of life, especially requirements for comfort and aesthetics, has also increased. Wearable antennas [13], due to their suitable environment and special working characteristics, also face many challenges, such as small size, good performance, low cost, and easy portability. Reference [14] proposes a wearable dual-band, dual-mode button antenna suitable for body communication, but the operating bandwidth of the antenna is only 250 MHz, and coaxial feeding has a significant impact on the antenna’s robust performance. Reference [15] proposes a conformal slot antenna with a bandwidth of approximately 450 MHz but a maximum gain of only 3 dBi. Reference [16] introduces a compact F-shaped slot monopole antenna. Although it has high gain, the antenna uses two materials and has a complex structure. Overall, most antennas can only meet a single performance index.

As wearable antennas are typically designed with a low profile while meeting the requirements of antenna miniaturization, the gain of the antenna will be greatly reduced. The corresponding interaction between the antenna and the human body will also weaken the performance of the antenna. How to reduce the impact between the antenna and the human body is also a key issue to be solved. With the emergence of metamaterial structures [17,18], new ideas have been provided to solve the comprehensive performance problems of antennas in order to ensure the stable operation of antennas and reduce the specific absorption rate (SAR) values for human safety.

In this approach, the SF-FDTD method is proposed, which selectively updates the H-field to retrieve accurate results. Updated equations for voltage sources and resistors are derived through WCS and HIE SF-FDTD methods. Numerical experiments verify the code, and stability and errors are analyzed. The results show good agreement with classical FDTD while significantly reducing computation time and memory usage. We design a dual-band monopole wearable antenna operating in a human local area network, and we use the characteristics of metamaterials to design a dual-ring, periodic, artificial magnetic conductor (AMC) [19] structure. Loading it below the antenna not only improves the gain but also ensures safety when acting on the human body. A practical example presents a layered broadband circularly polarized antenna with an impedance bandwidth of 2.4 GHz–2.48 GHz and 5.725 GHz–5.875 GHz, covering the ISM dual band [20]. The antenna is simulated using WCS and HIE SF-FDTD methods. To obtain better performance of the antenna, its size is optimized through artificial intelligence (AI). The fabricated prototype has been tested in our laboratory. The results show very good agreement between the experiment and the simulation. The main contributions of this paper are summarized as follows.

i. In order to refine the entire algorithmic system and further improve computational efficiency, a new SF-FDTD method is proposed based on the non-chiral Helmholtz time-domain equations, consisting of voltage and current sources. Resistance, inductance, and capacitance in lumped parameter circuits have been considered in the FDTD discrete region. The WCS and HIE SF-FDTD formula has been derived for general antennas, microwave circuits, and target scatterings.
ii. To validate our SF-FDTD code, a new dual-band wearable antenna, together with the AMC structure, are designed. These two structures are made of flexible materials. The antenna radiator and the AMC unit both use a double-ring structure to effectively reduce their size. It also achieves isolation of the antenna from the human body, ensuring the safety of electromagnetic radiation from wearable devices for the human body.
iii. To obtain the compact size of the antenna, we optimized the dimensions using the AI method. Through beam tracking optimization in wireless communications, an optimization scheme for coordinated beamforming is proposed to minimize interference between access points. Correlating these points with the sensitive dimensions of the antenna, the particle swarm optimization (PSO) method is used to find the effect of the dimensional change on the beam, and then optimization is performed to find the minimum value of this effect, which achieves minimization of the antenna’s dimensions.

The remainder of this paper is structured as follows. Section 2 derives the extended state–space model and formulates the problem for the SF-FDTD. Section 3 provides a detailed derivation of the antenna’s AMC design. Section 4 describes the joint simulation of the antenna and the AMC, and size optimization is presented in Section 5. Experimental results and the analysis are illustrated in Section 6. Finally, Section 7 concludes this paper.

## 2. Single-Field FDTD Method

Without generality, the updated equations for the 3D HIE and WCS SF-FDTD algorithms, including voltage sources and resistors, are derived from the time-domain Helmholtz wave equation. The lumped elements are added in the *z*-direction for HIE SF-FDTD and in the y-direction for WCS SF-FDTD. The relationship between the current density source *J*, voltage sources *V*, and resistors *R* is established as follows:

(1) $$J_{z}^{n+\frac{1}{2}}=\frac{\Delta z}{2\Delta x\Delta yR}\left(E_{z}^{n+1}+E_{z}^{n}\right)+\frac{1}{\Delta x\Delta yR}V_{s}^{n+\frac{1}{2}}$$

(2) $$J_{z}^{n-\frac{1}{2}}=\frac{\Delta z}{2\Delta x\Delta yR}\left(E_{z}^{n}+E_{z}^{n-1}\right)+\frac{1}{\Delta x\Delta yR}V_{s}^{n-\frac{1}{2}}$$

The basic updated equations for the 3D HIE and WCS SF-FDTD algorithms are introduced in [21,22]. Based on these, we derive the updated equations, including resistors *R* and voltage sources *V* for both methods. By updating the equations of the WCS SF-FDTD method, including voltage sources and resistors *R*, for the WCS SF-FDTD method, by discretizing Maxwell’s equations, we obtain

(3) $$H_{x}^{n+\frac{1}{2}}-H_{x}^{n-\frac{1}{2}}=\frac{\Delta t}{\mu }D_{z}E_{y}^{n}-\frac{\Delta t}{\mu }D_{y}E_{z}^{n}$$

(4) $$H_{y}^{n+\frac{1}{2}}-H_{y}^{n-\frac{1}{2}}=\frac{\Delta t}{\mu }D_{x}E_{z}^{n}-\frac{\Delta t}{\mu }D_{z}E_{x}^{n}$$

(5) $$H_{z}^{n+\frac{1}{2}}-H_{z}^{n-\frac{1}{2}}=\frac{\Delta t}{\mu }D_{y}E_{x}^{n}-\frac{\Delta t}{\mu }D_{x}E_{y}^{n}$$

as well as

(6) $$E_{y}^{n+1}-E_{y}^{n}=\frac{\Delta t}{\varepsilon }D_{z}H_{x}^{n+\frac{1}{2}}-\frac{\Delta t}{\varepsilon }D_{x}H_{z}^{n+\frac{1}{2}}-\frac{\Delta t\sigma_{y}^{e}}{\varepsilon }E_{y}^{n+\frac{1}{2}}-\frac{\Delta t}{\varepsilon }J_{y}^{n+\frac{1}{2}}$$

(6) Going one step back yields

(7) $$E_{y}^{n}-E_{y}^{n-1}=\frac{\Delta t}{\varepsilon }D_{z}H_{x}^{n-\frac{1}{2}}-\frac{\Delta t}{\varepsilon }D_{x}H_{z}^{n-\frac{1}{2}}-\frac{\Delta t\sigma_{y}^{e}}{\varepsilon }E_{y}^{n-\frac{1}{2}}-\frac{\Delta t}{\varepsilon }J_{y}^{n-\frac{1}{2}}$$

By applying the CN scheme to the field components operated by $D_{x}$ and $D_{z}$, we obtain

(8) $$H_{x}^{n+\frac{1}{2}}-H_{x}^{n-\frac{1}{2}}=\frac{\Delta t}{\mu }D_{z}\left(\frac{E_{y}^{n+1}+E_{y}^{n-1}}{2}\right)-\frac{\Delta t}{\mu }D_{y}E_{z}^{n}$$

(9) $$H_{z}^{n+\frac{1}{2}}-H_{z}^{n-\frac{1}{2}}=\frac{\Delta t}{\mu }D_{y}E_{x}^{n}-\frac{\Delta t}{\mu }D_{x}\left(\frac{E_{y}^{n+1}+E_{y}^{n-1}}{2}\right)$$

By subtracting (6) and (7), we obtain

(10) $$\begin{array}{c} E_{y}^{n+1}-2E_{y}^{n}+E_{y}^{n-1}=\frac{\Delta t}{\varepsilon }D_{z}\left(H_{x}^{n+\frac{1}{2}}-H_{x}^{n-\frac{1}{2}}\right)-\frac{\Delta t}{\varepsilon }D_{x}\left(H_{z}^{n+\frac{1}{2}}-H_{z}^{n-\frac{1}{2}}\right)\\ -\frac{\Delta t\sigma_{y}^{e}}{\varepsilon }\left(E_{y}^{n+\frac{1}{2}}-E_{y}^{n-\frac{1}{2}}\right)-\frac{\Delta t}{\varepsilon }\left(J_{z}^{n+\frac{1}{2}}-J_{z}^{n-\frac{1}{2}}\right) \end{array}$$

By substituting (8) and (9) into (10), we obtain

(11) $$\begin{array}{c} \left[1-\delta_{0}\left(D_{x}^{2}+D_{z}^{2}\right)\right]\left(E_{y}^{n+1}+E_{y}^{n-1}\right)\\ =2E_{y}^{n}-2\delta_{0}D_{y}D_{x}E_{x}^{n}-2\delta_{0}D_{y}D_{z}E_{z}^{n}-\beta_{y}\sigma_{y}^{e}\left(E_{y}^{n+\frac{1}{2}}-E_{y}^{n-\frac{1}{2}}\right)-\beta_{y}\left(J_{y}^{n+\frac{1}{2}}-J_{y}^{n-\frac{1}{2}}\right) \end{array}$$

By replacing $E_{\rho }^{n+\frac{1}{2}}$ and $E_{\rho }^{n-\frac{1}{2}}$ with $\left(E_{\rho }^{n+1}+E_{\rho }^{n}\right)/2$ and $\left(E_{\rho }^{n}+E_{\rho }^{n-1}\right)/2$, respectively, and substituting (1) into (11), we obtain

(12) $$\begin{array}{c} \left[1+\frac{\alpha_{y}\beta_{y}\Delta y}{2\Delta x\Delta zR}-\alpha_{y}\delta_{0}\left(D_{x}^{2}+D_{z}^{2}\right)\right]\left(E_{y}^{n+1}+E_{y}^{n-1}\right)\\ =2\alpha_{y}E_{y}^{n}+\left(\alpha_{y}\beta_{y}\sigma_{y}^{e}+\frac{\alpha_{y}\beta_{y}\Delta y}{\Delta x\Delta zR}\right)E_{y}^{n-1}-2\alpha_{y}\delta_{0}D_{y}D_{x}E_{x}^{n}-2\alpha_{y}\delta_{0}D_{y}D_{z}E_{z}^{n}\\ -\frac{\alpha_{y}\beta_{y}}{\Delta x\Delta zR}\left(V_{s}^{n+\frac{1}{2}}-V_{s}^{n-\frac{1}{2}}\right) \end{array}$$

To simplify this equation, we define $\gamma =\left(2\Delta x\Delta zR\right)/\left(2\Delta x\Delta zR+\alpha_{y}\beta_{y}\Delta y\right)$, and then we obtain

(13) $$\begin{array}{c} \left[1-\alpha_{y}\gamma \delta_{0}\left(D_{x}^{2}+D_{z}^{2}\right)\right]\left(E_{y}^{n+1}+E_{y}^{n-1}\right)\\ =2\alpha_{y}\gamma E_{y}^{n}+\left(\alpha_{y}\beta_{y}\gamma \sigma_{y}^{e}+\frac{\alpha_{y}\beta_{y}\gamma \Delta y}{\Delta x\Delta zR}\right)E_{y}^{n-1}-2\alpha_{y}\gamma \delta_{0}D_{y}D_{x}E_{x}^{n}-2\alpha_{y}\gamma \delta_{0}D_{y}D_{z}E_{z}^{n}\\ -\frac{\alpha_{y}\beta_{y}\gamma }{\Delta x\Delta zR}\left(V_{s}^{n+\frac{1}{2}}-V_{s}^{n-\frac{1}{2}}\right) \end{array}$$

Considering the Douglas Gunn (D-G) method [23], Equation (13) can be changed to

(14) $$\begin{array}{c} \left[1-\alpha_{y}\gamma \delta_{0}D_{x}^{2}\right]\left(E_{y}^{*}+E_{y}^{n-1}\right)\\ =2\alpha_{y}\gamma \left(1+\delta_{0}D_{z}^{2}\right)E_{y}^{n}+\left(\alpha_{y}\beta_{y}\gamma \sigma_{y}^{e}+\frac{\alpha_{y}\beta_{y}\gamma \Delta y}{\Delta x\Delta zR}\right)E_{y}^{n-1}\\ -2\alpha_{y}\gamma \delta_{0}D_{y}D_{x}E_{x}^{n}-2\alpha_{y}\gamma \delta_{0}D_{y}D_{z}E_{z}^{n}-\frac{\alpha_{y}\beta_{y}\gamma }{\Delta x\Delta zR}\left(V_{s}^{n+\frac{1}{2}}-V_{s}^{n-\frac{1}{2}}\right) \end{array}$$

and

(15) $$\left(1-\alpha_{y}\gamma \delta_{0}D_{z}^{2}\right)\left(E_{y}^{n+1}-2E_{y}^{n}+E_{y}^{n-1}\right)=E_{y}^{*}-2E_{y}^{n}+E_{y}^{n-1}$$

By introducing auxiliary variables $e_{y}^{*}=E_{y}^{*}+E_{y}^{n-1}$ and $e_{y}^{n+1}=E_{y}^{n+1}-2E_{y}^{n}+E_{y}^{n-1}$, we finally obtain

(16) $$\begin{array}{c} \left[1-\alpha_{y}\gamma \delta_{0}D_{x}^{2}\right]e_{y}^{*}=2\alpha_{y}\gamma \left(1+\delta_{0}D_{z}^{2}\right)E_{y}^{n}+\left(\alpha_{y}\beta_{y}\gamma \sigma_{y}^{e}+\frac{\alpha_{y}\beta_{y}\gamma \Delta y}{\Delta x\Delta zR}\right)E_{y}^{n-1}\\ -2\alpha_{y}\gamma \delta_{0}D_{y}D_{x}E_{x}^{n}-2\alpha_{y}\gamma \delta_{0}D_{y}D_{z}E_{z}^{n}-\frac{\alpha_{y}\beta_{y}\gamma }{\Delta x\Delta zR}\left(V_{s}^{n+\frac{1}{2}}-V_{s}^{n-\frac{1}{2}}\right) \end{array}$$

and

(17) $$\left(1-\alpha_{y}\gamma \delta_{0}D_{z}^{2}\right)e_{y}^{n+1}=e_{y}^{*}-2E_{y}^{n}$$

From the equations above, the coefficients are defined as $\delta_{0}=\Delta t^{2}/2\mu_{0}\varepsilon_{y}$, $\beta_{y}=\Delta t/\varepsilon_{y}$, and $\alpha_{y}=2/\left(2+\sigma_{y}^{e}\beta_{y}\right)$. The updated equations of the HIE SF-FDTD method include the voltage sources and the resistors.

Next, we present the HIE SF-FDTD updated equations including these parameters. Hereinafter, the parameters in the coefficients are defined as $a_{z}=\sigma_{z}^{e}\left(i,j,k\right)/2+\varepsilon /\Delta t$, $b_{z}=\sigma_{z}^{e}\left(i,j,k\right)/2-\varepsilon /\Delta t$, γ′=2ε/Δt, and δ=Δt/2μ, while auxiliary variables are introduced as $e_{x}^{n+1}=E_{x}^{n+1}+E_{x}^{n-1}$ and $e_{y}^{n+1}=E_{y}^{n+1}+E_{y}^{n-1}$. Through the HIE SF-FDTD method, we obtain

(18) $$\begin{array}{c} E_{z}^{n+1}=C_{ez1}E_{z}^{n}+C_{ez2}\left(D_{x}^{2}+D_{y}^{2}\right)E_{z}^{n}+C_{ezm1}E_{z}^{n-1}-C_{ex}D_{z}D_{x}e_{x}^{n+1}-C_{ey}D_{z}D_{y}e_{y}^{n+1}\\ -C_{s}\left(V_{s}^{n+\frac{1}{2}}-V_{s}^{n-\frac{1}{2}}\right) \end{array}$$

For the updated equation, including the voltage source with internal resistance *R*, $C_{ez1}=2\gamma \prime \Delta x\Delta yR/A$, $C_{ez2}=4\delta \Delta x\Delta yR/A$, $C_{ezm1}=\left(2b_{z}\Delta x\Delta yR+\Delta z\right)/A$, $C_{ex}=2\delta \Delta x\Delta yR/A$, $C_{ey}=2\delta \Delta x\Delta yR/A$, and $C_{s}=2/A$, where $A=2a_{z}\Delta x\Delta yR+\Delta z$.

Note that in order to obtain the updated equations, including the resistor *R*, we can simply set the coefficient before the voltage source term as zero. The updated equations for other lumped components, such as capacitor *C* and inductor *L*, can be derived in a similar manner.

To benchmark our SF-FDTD method, we compared its accuracy and computational efficiency against classical FDTD as well as modern unconditionally stable methods, such as ADI-FDTD [5] and Newmark-beta FDTD [6]. As shown in Section 5, the SF-FDTD achieves comparable accuracy and a mean absolute percentage error (*MAPE* < 2.5%) similar to classical FDTD while reducing memory usage by 30% and simulation time by 20%. Unlike ADI-FDTD, which introduces splitting errors due to field decoupling, our method selectively updates H-fields only where necessary (e.g., near lumped components), avoiding artificial dispersion in multi-scale problems.

To check our code according to the formulas above, there are some real examples, such as microwave circuits, antennas, and target scattering. However, the new designs are more challenging. We have designed a new antenna based on the needs of medical testing instruments, which will be used in wearable devices. The design procedure is illustrated as follows.

## 3. Antenna with AMC Enhanced Structure Design

For the wearable devices, the proposed antenna uses a flexible material, which consists of two layers, fed by a coplanar waveguide. The top layer is a double-locked ring monopole antenna, and the bottom layer is a double-layered artificial magnetic conductor structure. Both structures use a polyimide (PI) dielectric substrate with a dielectric constant of 3.4 and a thickness of 0.3 mm. To support them, the air gap between the antenna and the AMC is filled with Teflon (*ε_r_* ≈ 1) instead.

**Figure 1:**
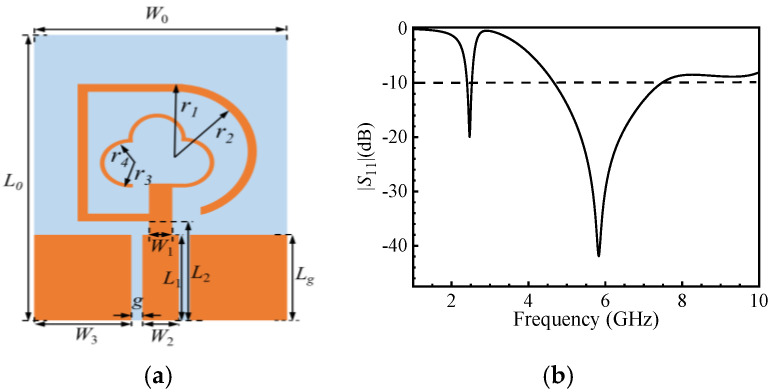
The custom required antenna with flexible and dual-band properties; (**a**) structure with two open-loop monopoles fed by a coplanar waveguide; (**b**) |*S*_11_| parameters from initial simulation.

The dimensional size design of the monopole antenna, as shown in Figure 1a, consists of two bent branches forming inner and outer rings. The outer ring generates a low-frequency band at 2.45 GHz, while the inner ring generates a high-frequency band at 5.8 GHz. These frequencies, as shown in Figure 1b, were initially obtained using our code.

**Figure 2:**
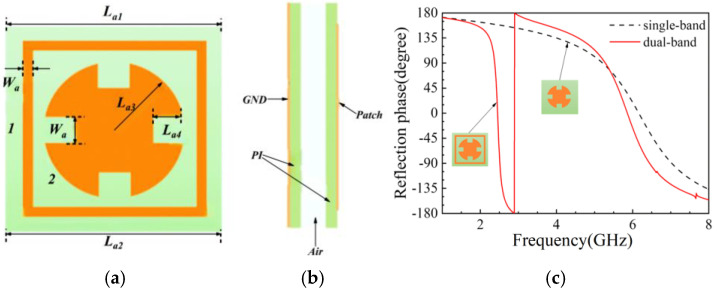
One unit of the AMC structure zoomed in with a dual-band property; (**a**) top view and dimensional size in detail; (**b**) side view showing the air gap between the antenna and the AMC; (**c**) reflection phase displaying the newly designed AMC with dual-band characteristics.

For the wearable antenna, electromagnetic safety must be considered. Therefore, we design an AMC structure together with the antenna. It consists of a 3 × 3 element array. Each unit of the AMC structure, as shown in Figure 2a, achieves dual resonance with a reflection phase bandwidth of 2.38–2.54 GHz, as shown in Figure 2b. The simulated result is also obtained using our code above.

**Figure 3:**
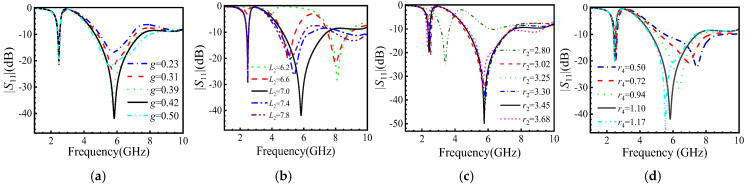
Simulated |*S*_11_| of the antenna shown in Figure 1 related to the main size (unit: mm) changing, with (**a**) *g*, (**b**) *L*_2_, (**c**) *r*_2_, and (**d**) *r*_4_. We can choose the best sizes for meeting the requirements.

To check the stability when the antenna size changes, we scan the main size of designed antenna, as shown in Figure 1. We can choose the best size matching. Simulated results of the |*S*_11_| are shown in Figure 3. The antenna achieves a good impedance bandwidth when the gap g between the ground plane and the feeder is 0.42 mm and the feeder length *L*_2_ is 7 mm. The size of *r*_2_ significantly affects the low-frequency band, while *r*_3_ and *r*_4_ affect the high-frequency band. The antenna operates between 4.66 GHz and 7.48 GHz when *r*_4_ = 1.1 mm. These sizes can be used in the model analysis.

## 4. Joint Simulation and Robustness Analysis

**Figure 4:**
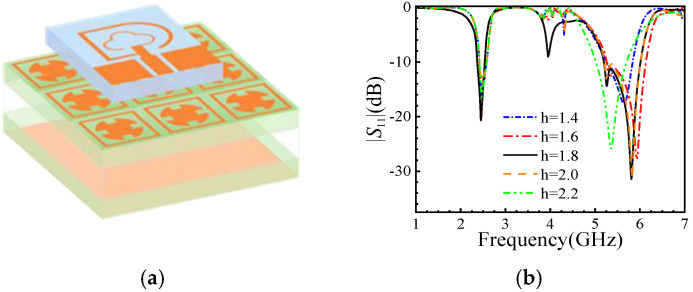
Joint simulation of the antenna structure and the AMC; (**a**) schematic diagram; (**b**) |*S*_11_| variation with distance *h* between the antenna and the AMC, where *h* = 1.8 mm found the best size.

To save simulation time, an AMC structure consists of 3 × 3 units, as shown in Figure 4a. It is put into the three-layered skin model [24]. This AMC structure can be used to reduce the high-frequency bandwidth, but it maintains the low-frequency bandwidth. Figure 4b shows that the antenna’s performance remains stable with varying distances *h* between the antenna and the AMC structure. From Figure 4b, it is shown that the additional structure can enhance the antenna’s gain, and around typical frequencies of 2.45 GHz and 5.8 GHz, it remained stable.

At *h* = 1.8 mm, the antenna achieves peak gain (8.9 dBi) and minimal SAR (1.2 W/kg). Increasing *h* to 5 mm reduces the gain to 7.1 dBi while raising the SAR to 1.8 W/kg, indicating a trade-off between safety and performance.

To further validate the SF-FDTD method, we compared key parameters (*S*_11_, gain) using CST Microwave Studio (Ver. 2019) simulations. Discrepancies (<3%) primarily stem from meshing differences (SF-FDTD uses uniform grids vs. CST’s adaptive meshing). While commercial tools offer higher precision for small-scale structures, the SF-FDTD’s computational efficiency, as shown in Section 5, makes it preferable for large-scale AMC–antenna co-simulations.

**Figure 5:**
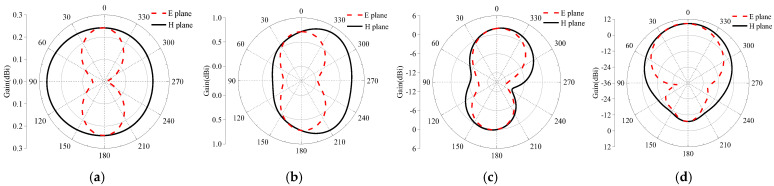
Radiation pattern of designed antenna without and with AMC structure; (**a**,**b**) the single antenna at 2.45G Hz and 5.8 GHz, respectively; (**c**,**d**) the antenna together with the AMC structure at 2.45 GHz and 5.8 GHz, respectively.

The radiation pattern is calculated, as shown in Figure 5, at a frequency point of 2.45 GHz and 5.8 GHz. For the single antenna without AMC, the results are depicted in Figure 5a,b. It displayed good omnidirectional results. With AMC, the results are shown in Figure 5c,d. Based on a comparison of Figure 5a,c, it is evident that after adding the AMC structure, the forward gain at 2.45 GHz increases from −3.7 dBi to 1.78 dBi, and the forward gain at 5.8 GHz changes from omnidirectional to directional. Based on a comparison of Figure 5b,d, the gain also increases from −0.3 dBi to 8.8 dBi, above 5 dBi. Therefore, the AMC structure can effectively improve the antenna’s gain.

**Figure 6:**
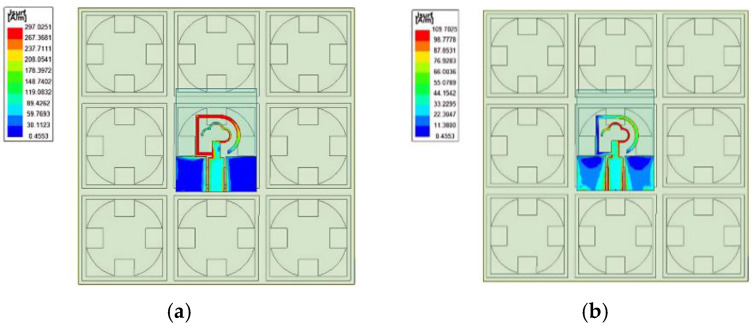
Vector surface current distribution of the antenna with AMC structure at (**a**) 2.45 GHz and (**b**) 5.8 GHz.

The current distribution on the antenna’s surface is simulated at 2.45 GHz and 5.8 GHz, as shown in Figure 6. By comparing the current distribution at two frequency points, the similarity is that the current strength is mainly concentrated near the feed line and near the ground plane. The difference is that the electric field strength positions on the radiation patch are different. The low-frequency part is mainly concentrated in the left closed-loop part, and the high-frequency part is mainly concentrated in the right open-loop part. By analyzing the current’s direction, the relative position between the antenna and the AMC is adjusted, and the antenna performs best when placed in a center up position.

**Figure 7:**
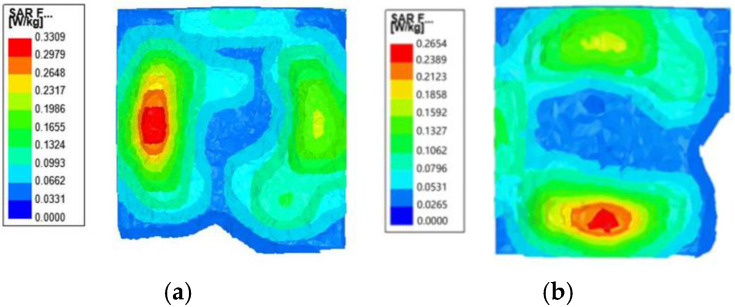
The specific absorption rate (SAR) value distribution on a human body at (**a**) 2.45 GHz and (**b**) 5.8 GHz.

Then, we establish a three-layer skin simulation model, put the antenna and the antenna loaded with the AMC structure into the simulation environment, and simulate the SAR values of the two structures in the simulation environment. The results, as shown in Figure 7, prove that the SAR values of both frequency bands can be reduced to below the safety standard of 1.6 W/kg after loading the AMC structure, which meets the application requirements.

**Figure 8:**
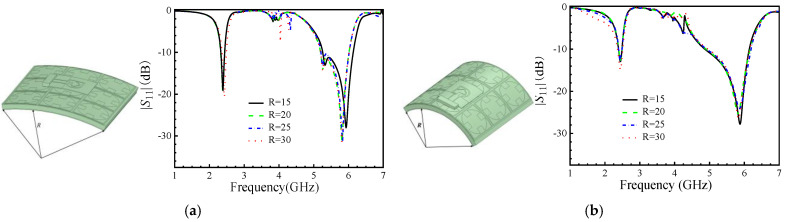
The |*S*_11_| while the antenna’s dielectric substrate is bent in different directions in different radiuses R (unit: mm); (**a**) bent along with the length directions; (**b**) bent along with the width of directions.

When the antenna is conformal to the human body, there will be different degrees and directions of bending, as shown in Figure 8. Different radius cylinders are used to simulate the bending situation, and the antenna is bent along with the length and width directions of the dielectric substrate as arcs. Figure 8 shows the |*S*_11_| after bending at different radiuses. The simulation was conducted with radiuses of 15 mm, 20 mm, 25 mm, and 30 mm. The results show that the bending in the length and width directions has almost the same effect on the low-frequency region, while slight deviations occur in the high-frequency region. As the curvature of the bending changes, the antenna can always maintain a stable operating bandwidth in the corresponding frequency bands.

**Figure 9:**
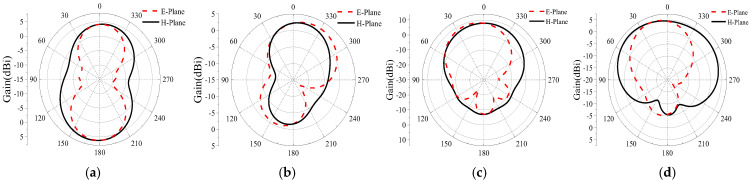
Radiation pattern of antennas with different bending radiuses; (**a**) R = 15 mm and (**b**) R = 35 mm at 2.45 GHz, as well as (**c**) R = 15 mm and (**d**) R = 35 mm at 5.8 GHz.

The radiation patterns of the bent antenna have been simulated at two frequency bands, 2.45 GHz and 5.8 GHz, as depicted in Figure 9. When the bent radius R = 35 mm, it is almost unchanged compared to the antenna without bending. However, when the R = 15 mm, as the curvature deepens, the radiation pattern of the antenna changes to a certain extent and is accompanied by slight distortion.

## 5. AI Powered the Antenna Size’s Optimization

From Figure 3, Figure 4 and Figure 8, we have used the traditional method to scan the relative size of the antenna to find the best size. This method helps us decide. It has been proven that the efficiency is a bit lower. Therefore, we must attempt to find these optimal parameters using today’s popular AI method, the particle swarm optimization (PSO) algorithm [25].

We were inspired by beam tracking optimization in wireless communications. An optimization scheme for coordinated beamforming is proposed to minimize interference between access points. The problem is formulated as a mutual interference optimization scheme for coordinated beamforming. To accurately express the problem and solve it, the following symbols are defined. First, *K* represents the number of access points. A two-dimensional array *X* is introduced to represent the pairing relationship between access points, where the value of *X_I,k_* = 1 if access points *i* and *k* in the same frequency domain have a clustering relationship; otherwise, *X_I,k_* = 0. To simplify the notation without losing generality, it is assumed that each access point serves only one station when performing coordinated beamforming. Thus, the channel matrix composed of each access point *i* and its associated station can be decomposed using singular value decomposition, as $H_{i}=U_{i}\Sigma_{i}V_{i}^{H}$. $x_{i}={\left[x_{I,1}x_{I,2}\ldots x_{I,N_{tx}}\right]}^{T}$ is defined as the baseband data *N_tx_* at the transmitter with $y_{i}={\left[y_{I,1}y_{I,2}\ldots y_{I,N_{rx}}\right]}^{T}$ data streams, as the baseband data at the receiver with *N_rx_* data streams, and $n_{i}={\left[n_{I,1}n_{I,2}\ldots n_{I,N_{rx}}\right]}^{T}$ as the Gaussian white noise at the receiver. According to the coordinated beamforming process, each access point can obtain the channel state information of another access point. Therefore, we have four channel matrices, $H_{I},H_{k},H_{I,k},H_{k,i}$, where Hi represents the channel matrix from access point i to its associated station and *H_I,k_* represents the channel matrix from access point i to access point *k*, which is the channel matrix experienced by the interference signal. Thus, the receiver expression is given by

(19) $$\begin{array}{c} y_{i}^{\prime }=U_{i}^{H}\left(\sum_{k=1}^{K}H_{k}V_{k}x_{k}\right)+U_{i}^{H}n_{I}\\ =U_{i}^{H}\left(U_{i}\Sigma_{i}V_{i}^{H}\right)V_{i}x_{i}+U_{i}^{H}\sum_{k\neq i}^{K}\left(U_{k,i}\Sigma_{k,i}V_{k,i}^{H}\right)V_{k}x_{k}+U_{i}^{H}n_{i} \end{array}$$

On the other hand, coordinated beamforming requires minimizing the interference between access points. This necessitates that the interference beamforming matrix falls within the null space of the equivalent channel matrix experienced by the interference signal, i.e., $V_{k}\in Null\left({U_{i}^{H}H}_{k,i}\right)$. If the number of transmitting antennas is sufficient to fully fall within the null space (completely eliminating interference in the transmission of access point i and achieving perfect parallel transmission), then the primary objective in this scenario is to find the beamforming matrix that maximizes the throughput of access point *k*. We can formulate optimization problem 1 as follows.

(20) $$\begin{array}{c} {\mathrm{argmin}}_{w}\left\|\tilde{P}-V_{i}\right.\|s.t.\tilde{P}\in \mathrm{Null}\{U_{i}^{H}H_{k,i}\},\\ {V_{i}}^{H}V_{i}={V_{i}V_{i}}^{H}=I={\tilde{P}}^{H}\tilde{P}=\tilde{P}{\tilde{P}}^{H} \end{array}$$

Currently, a suboptimal solution is the Zero-Forcing (ZF) method or Block Diagonalization (BD). The specific process involves finding any unitary matrix ${\tilde{P}}_{i}\in \mathrm{Null}\{U_{i}^{H}H_{I,k}\}$ and ${\tilde{P}}_{k}\in \mathrm{Null}\{U_{i}^{H}H_{k,i}\}$ in the null space and then defining the beamforming matrices as ${{\tilde{V}}_{i}={\tilde{P}}_{i}\left(V_{i}^{H}{\tilde{P}}_{i}\right)}^{-1}$ and, similarly, ${{\tilde{V}}_{k}={\tilde{P}}_{k}\left(V_{k}^{H}{\tilde{P}}_{k}\right)}^{-1}$. This results in

(21) $$y_{i}^{\prime }=U_{i}^{H}H_{i}{\tilde{V}}_{i}x_{i}+U_{i}^{H}H_{k,i}{\tilde{V}}_{k}x_{k}=\Sigma_{i}x_{i}$$

Therefore, in this case, it is possible to perfectly eliminate transmission interference while maintaining the performance of the original singular value decomposition (SVD). However, another possibility is that an insufficient number of transmitting antennas may prevent the signals from fully falling into the null space. In such a scenario, it is necessary to consider either reducing the number of data streams or implementing partial interference beamforming matrices. In this case, we can formulate optimization problem 2 as follows.

(22) $$\begin{array}{c} {\mathrm{argmin}}_{\tilde{P}}\left\|\tilde{P}-V\right.\|s.t.V\in \mathrm{Null}\{U_{i}^{H}H_{k,i}\}\\ {V_{i}}^{H}V_{i}={V_{i}V_{i}}^{H}=I={\tilde{P}}^{H}\tilde{P}=\tilde{P}{\tilde{P}}^{H} \end{array}$$

We can utilize the properties of unitary matrices, matrix theory, and the diverse service requirements in collaborative beamforming, such as stream number control or transmission rate, to conduct a categorized analysis. Ultimately, this approach allows us to derive optimal solutions under different quality-of-service (QoS) requirements.

The PSO algorithm utilized 50 particles and 100 iterations, with a convergence criterion of <1% change in fitness (*S*_11_ minimization) over 10 consecutive iterations. Each fitness evaluation involved a parallelized SF-FDTD simulation, requiring approximately 8 h on a 16-core CPU. Sensitivity analysis revealed that parameters *r*_2_ (outer ring radius) and *L*_2_ (feeder length) exhibited the strongest correlation with the bandwidth (R^2^ > 0.85), guiding the final optimization trajectory. Key optimized parameters (Table 1) were derived via sensitivity analysis, where *r*_2_ and *L*_2_ exhibited the strongest correlation with the bandwidth. The PSO “fitness” function is as follows.

Fitness = Σ|*S*_11_(fi)|^2^, where fi ∈ [2.4,2.48] ∪ [5.725,5.875] (23)

The optimization procedure is implemented as follows:

Initialize particles with random positions/speeds.

      For each iteration:

       Run SF-FDTD simulations in parallel;

       Calculate fitness (*S*_11_ minimization);

       Update particle velocities/positions;

       Check convergence (Δfitness < 1% for 10 steps);

      Output optimized dimensions (Table 1).

Each channel matrix expresses an unknown size of the designed antenna. We can solve the required optimal size, as listed in Table 1.

In addition to the shorter computation time, the optimization method for the above dimensions is different from the principle of our proposed FDTD method in that the objective Function (22) can be approximated to reach the optimum through multiple approximations, which is still not intuitive enough in comparison with the traditional method. Therefore, it is presented here as a mere attempt to try a new method.

It is worth mentioning that in the SF FDTD method, we do not have to iterate the magnetic field components at all positions. Only the H fields needed to calculated the sampled currents are updated. The total time consumption of the proposed method in the above experiments and a comparison with the classical FDTD method is shown in Table 2. Simulations were performed on a 16-core CPU (Intel Xeon Gold 6248R, in Dell personal computer, made in China, Tianjin) with uniform mesh density of λ/20 at 5.8 GHz. Time savings are consistent across mesh densities (λ/10 to λ/30).

We define the mean absolute percentage error (*MAPE*) in the S parameters as

(24) $$MAPE=\frac{100}{N}\sum_{n=1}^{N}\left|\frac{S_{SF}\left(n\right)-S_{FDTD}\left(n\right)}{S_{FDTD}}\right|$$

The *MAPE* compared to the classical FDTD method is listed in Table 3. From the tables, we find that the HIE SF-FDTD method saves a higher percentage of time, but its precision is lower than that of the WCS SF-FDTD method, which saves a relatively lower percentage of time.

## 6. Physical Process Testing

**Figure 10:**
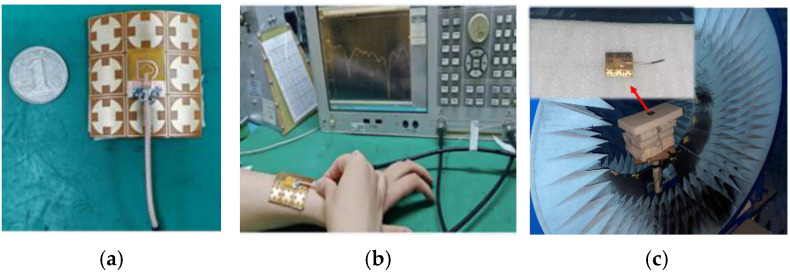
Physical antenna and test scenarios; (**a**) antenna with AMC; (**b**) vector network analyzer testing while the antenna was placed on the forearm of the volunteer; (**c**) microwave anechoic chamber environment testing.

**Figure 11:**
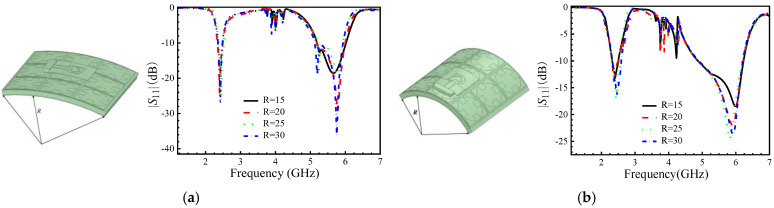
Actual measurement of antenna |*S*_11_| when bending, along with (**a**) substrate length and (**b**) substrate width.

To check the antenna’s performance, a fabricated sample has been tested in our laboratory by using a vector network analyzer, and a microwave anechoic chamber(SiYi, AV3656, made in China, Qingdao). The antenna was placed 5 mm from the forearm and tested in a shielded chamber. The results, as shown in Figure 10, demonstrate stable performance even when the antenna is bent. The measured results closely match the simulations, confirming the antenna’s robustness. The |*S*_11_| parameters of the antenna structure were tested using a vector network analyzer, and the far-field radiation pattern of the antenna was tested in a microwave anechoic chamber environment, as shown in Figure 11. When the antenna was simulated on the surface of the human body, bending simulation tests were conducted in different directions and at different radiuses. After bending in the length and width directions of the substrate, the bandwidth was slightly reduced compared to not bending, and it can work normally in the required dual-frequency band.

From the test results in Figure 11, we find that when the bending degree increases, the center frequency point of the antenna |*S*_11_| at high frequencies shifts, and there is a certain degree of deformation compared to the original simulation results, which may be caused by operational errors, such as placement and machining after loading the AMC structure, as well as coupling effects between the two structures, which are generally within an acceptable range.

**Figure 12:**
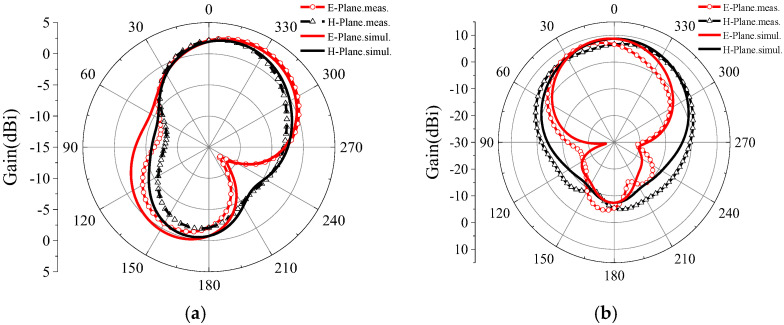
Radiation pattern measured and simulated at (**a**) 2.45 GHz and (**b**) 5.8 GHz.

Figure 12 shows the radiation pattern of the antenna in a microwave anechoic chamber at 2.45 GHz and 5.8 GHz. Upon comparison, it is found that there was almost no difference between the measured and simulated results, and the antenna’s performance was stable.

The SF-FDTD method accurately predicts far-field characteristics, including a 3 dB beamwidth of 78° at 5.8 GHz, which matches the experimental results within a 2° margin. Additionally, the calculated directivity (8.2 dBi at 5.8 GHz) aligns with the measurements (8.1 dBi), confirming the method’s fidelity in complex near-to-far-field transformations.

Compared to recent wearable antennas [13,14,15,16], as shown in Table 4, our design achieves superior gain (5.3 dBi/8.9 dBi vs. typical 3–5 dBi) and bandwidth (250 MHz vs. 450 MHz in [15]) while maintaining a compact size (15.6 × 20 mm^2^).

The proposed SF-FDTD code is compared with commercial CST software, (Ver. 2019), Frequency Domain Solver, −30 dB accuracy, and the results are listed in Table 5. At 5.8 GHz, we found that the percentage error is very small.

Discrepancies arise primarily from meshing differences, yet the results align within 3%, validating the SF-FDTD’s reliability. Commercial tools were not used extensively due to computational resource constraints for large-scale AMC–antenna co-simulations.

To assess generality, simulations were repeated on a five-layer heterogeneous model (skin–fat–muscle–bone–tissue). The AMC maintained SAR < 1.6 W/kg across all layers, with less than 5% deviation in *S*_11_ compared to the homogeneous model. Future experimental validation will employ anatomically realistic phantoms to account for tissue variability.

## 7. Conclusions

The proposed SF-FDTD method reduces simulation time and memory usage while maintaining accuracy. The designed antenna achieves dual-band and lower SAR performance, making it suitable for wearable wireless communication applications. Future work will focus on reducing antenna size and improving gain. The designed antenna was fed by a coplanar waveguide and AMC. The technology can effectively solve the problems of big size and radiation direction. By loading the AMC structure, we greatly improve the gain of the antenna and its isolation from the human body. Bending performance tests were conducted on the designed antenna, and the results showed good robustness. This designed wearable antenna has a small enough size and high gain, meeting the requirements of the ISM communication frequency band. From the experimental results in Section 6, we find that the simulation results in our code are in very good agreement with the tests. Both the algorithm and the antenna design have achieved the custom requirements.

While the dual-ring AMC structure effectively enhances isolation and gain, future designs could integrate reconfigurable intelligent surfaces (RIS) or holographic meta-surfaces [26,27,28]. For instance, the 2-bit RIS in [27] enables dynamic wavefront manipulation through rapid-response phase control, potentially optimizing antenna performance across varying body positions. However, such approaches may require additional control circuitry and complex fabrication, contrasting with the simplicity of our static AMC. Further studies should explore trade-offs between reconfigurability, computational overhead, and practical feasibility in wearable applications.

Integrating electromagnetic shielding layers (e.g., conductive textiles) could further reduce SAR. Preliminary simulations show a 30% SAR reduction when a 0.1 mm graphene layer is added, albeit with a 0.8 dB gain penalty. Balancing safety and performance will be explored in subsequent studies.

Future work will explore integrating electromagnetic shielding layers (e.g., conductive textiles or graphene films) to further reduce SAR. Preliminary simulations indicate that a 0.1 mm graphene layer between the AMC and the human body reduces SAR by 30%, albeit with a 0.8 dB gain penalty. Balancing safety enhancements with performance trade-offs will be critical for next-generation wearable devices.

## Figures and Tables

**Table 1:** Geometrical sizes for proposed antenna after optimization (unit: mm).

Dimension	*L*_0_	*W*_0_	*H*	*W*_1_	*L*_1_	*L*_2_	*W*_2_
Size	20	15.6	0.3	1.5	6.6	7	3
Dimension	*W*_3_	*r*_1_	*r*_2_	*r*_3_	*r*_4_	*L_g_*	*g*
Size	6	3.95	3.3	1.46	1.1	6.8	0.3
Dimension	*L_a_*_1_	*L_a_*_2_	*L_a_*_3_	*L_a_*_4_	*W_a_*_1_	*W_a_*_2_	*H*_l_
Size	17.27	16.5	5.7	3.46	0.42	3.8	2.2

**Table 2:** Comparison of simulation time with classical FDTD from Figure 9a,b at 2.45 GHz only.

Bent radius (mm)	R = 15	R = 35
Proposed method(s)	188	217
Classical FDTD(s)	238	268
Percentage of time saved	21.0%	19.0%

**Table 3:** MAPE of obtained parameters |*S*_11_| at different frequency bands compared with classical FDTD.

Bent radius (mm)	R = 15	R = 35
MAPE at 2.45 GHz	0.22%	2.2%
MAPE at 5.8 GHz	0.54%	1.9%

**Table 4:** Antenna performance table.

Metric	This Work	[14]	[15]
Peak Gain (dBi)	8.9	3	3.5
Bandwidth (MHz)	450	250	450
SAR (W/kg)	1.2	1.8	2

**Table 5:** Comparing SF-FDTD results with CST Microwave Studio simulating the design above; both have the same grid size.

Parameter	SF-FDTD (5.8 GHz)	CST (5.8 GHz)	Error (%)
|*S*_11_| (dB)	−25.2	−24.8	1.6
Peak Gain (dBi)	8.9	8.7	2.3
Radiation Efficiency (%)	60	61.5	2.5
Simulation Time (min)	31	37	

## Data Availability

The original contributions presented in the study are included in the article, further inquiries can be directed to the corresponding author.
